# A Cardiac Magnetic Resonance Study: Comparison of Biventricular Longitudinal Function in Hypertrophic Cardiomyopathy Patients and Normal Individuals

**DOI:** 10.7759/cureus.34165

**Published:** 2023-01-24

**Authors:** Özge Özden Tok, Gulsum Bingol, Serkan Unlu, Ferit Boyuk

**Affiliations:** 1 Cardiology, Memorial Bahçelievler Hospital, Istanbul, TUR; 2 Cardiology, Memorial Bahcelievler Hospital, Istanbul, TUR; 3 Cardiology, Gazi University Medical Faculty, Ankara, TUR; 4 Cardiology, Yedikule Chest Diseases and Thoracic Surgery Training and Research Hospital, Istanbul, TUR

**Keywords:** heart failure, tapse, mapse, cardiac mri, hypertrophic cardiomyopathy

## Abstract

Objective: Hypertrophic cardiomyopathy (HCM) is a genetic disease with an incidence of 0.2%-0.5%. It has a wide range of clinical presentations varying from coincidental diagnoses to heart failure, ventricular arrhythmias and sudden cardiac death. Mitral annular plane systolic excursion (MAPSE) and tricuspid annular plane systolic excursion (TAPSE) are M-mode-derived practical and reproducible measurements of systolic longitudinal displacement of the annular plane. These two measures may be used as markers of the left ventricular and right ventricular longitudinal functions. Currently, there are only a few studies on cardiac magnetic resonance (CMR)-derived TAPSE and MAPSE measurement comparison between the HCM group and normal control group. The aim of our study is to show the differences in CMR-derived TAPSE and MAPSE values between the HCM and normal population.

Methods: We evaluated CMR exams of patients diagnosed with HCM and of normal individuals scanned between 2020 and 2021 retrospectively. The patients were from our own institution’s and other hospitals’ in- and out-patient departments. Data was collected on 36 HCM patients and 34 adults with no known history of cardiac and non-cardiac diseases. All CMR exams were performed on a 1.5 T (Magnetom Avanto, Siemens Healthcare, Erlangen, Germany) scanner. CMR-derived MAPSE and TAPSE were measured on standard four-chamber steady-state free precession (SSFP) cine images and given in millimeters.

Results: From February 2020 to December 2021, a total of 150 patients were diagnosed with hypertrophic cardiomyopathy. After exclusion, 36 patients with HCM were included in the study and the normal control group comprised 34 individuals. The mean age of the HCM group was 43.2 + 13.5 years, while it was 37.5 + 11.3 in the control group. The female ratio of the HCM group was found to be 36%, while it was 56% in the healthy control group. MAPSE values were significantly higher in the normal control group when compared to the HCM patient group (MAPSE: 14.5 ± 2.9 mm vs. 11.7 ± 3.2 mm; p<0.001), while TAPSE values did not depict a significant difference between the two groups (p=0.627).

Conclusions: This study suggests that MAPSE values are significantly lower in the HCM patient group in comparison with the normal control group on CMR scans. Although not statistically significant, TAPSE values are also lower in the HCM group.

## Introduction

Hypertrophic cardiomyopathy (HCM) is a genetic disease with an incidence of 0.2%-0.5% in the general population [1-3]. It has a wide range of clinical presentations varying from asymptomatic coincidental diagnoses to heart failure, ventricular arrhythmias and sudden cardiac death [4,5]. Mitral annular plane systolic excursion (MAPSE) and tricuspid annular plane systolic excursion (TAPSE) are M-mode-derived quick, practical, sensitive and reproducible measurements of systolic longitudinal displacement of the annular plane [6-8]. These two indices may therefore be used as markers of the left ventricular and right ventricular longitudinal functions [7,9], and it has been well-documented that these parameters are in correlation with the ventricular ejection fraction [10-12]. Currently, there are only scarce data on cardiac magnetic resonance (CMR)-derived TAPSE and MAPSE measurement comparison between the HCM patients and normal control group. The aim of the current study is to demonstrate the differences in CMR-derived TAPSE and MAPSE values between the HCM and normal population.

## Materials and methods

**Figure 1:**
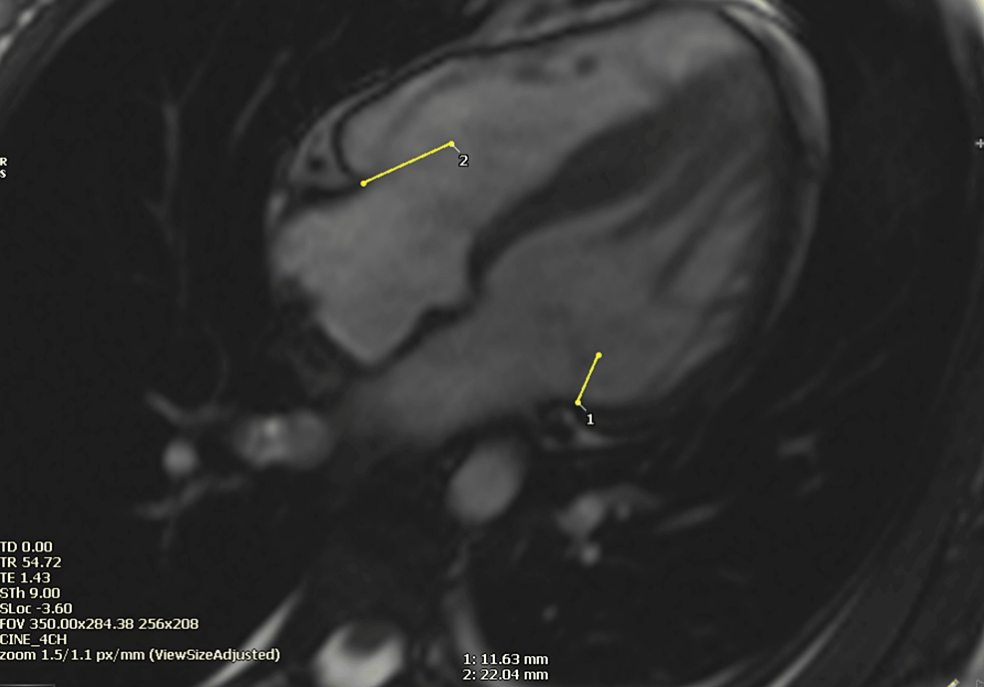
Measurement of the distance as a result of the motion of tricuspid and mitral annulus motion between end-diastolic and end-systolic phases on SSFP cine four-chamber image SSFP: steady-state free precession. Yellow arrows indicate (1) MAPSE: mitral annular plane systolic excursion and (2) TAPSE: tricuspid annular plane systolic excursion.

After our local ethic committee approval, we evaluated CMR exams of patients diagnosed with HCM and of the normal individuals scanned between 2020 and 2021 retrospectively. The patients were referred from our own institution’s and other hospitals’ in- and out-patient departments with various indications. Data was collected on 36 HCM patients and 34 adults with no known history of cardiac and non-cardiac diseases referred for cardiac magnetic resonance (CMR) imaging with indications such as bad echogenicity, palpitation, family history of different cardiomyopathies and normal CMR findings at the end. HCM patients were included when sarcomeric genes were identified with left ventricular hypertrophy >13 mm or in the absence of a mutation if LVH >15 mm with no other underlying reason. HCM patients were excluded if they had a systemic disease, an LVEF <55%, coronary artery disease, hypertension, chronic kidney disease, pacemakers or defibrillators and valvular disease that is more than mild. All CMR exams were performed on a 1.5 T (Magnetom Avanto, Siemens Healthcare, Erlangen, Germany) scanner at Memorial Bahçelievler Hospital. Cine images were acquired using standard methods. TAPSE, MAPSE, left ventricle (LV) and right ventricle (RV) volumes, function, LV myocardial mass and maximum segmental wall thickness, late gadolinium enhancement (LGE) and T1 map values were analyzed with CVI 42© (Circle Cardiovascular Imaging, Calgary, Canada). All the CMR scans were evaluated by a CMR-trained and experienced cardiologist. CMR-derived MAPSE and TAPSE were measured on standard four-chamber steady-state free precession (SSFP) cine images twice. The distance as a result of the motion of tricuspid and mitral annulus motion between end-diastolic and end-systolic phases was measured as shown in Figure 1. The TAPSE and MAPSE measurements were given in millimeters.

Statistical analysis

Continuous variables are presented as mean ± standard deviation, and categorical data are presented as percentages or frequencies. Continuous variables were examined by the Kolmogorov-Smirnov test to check for the normality of distribution. Student t-test and Mann-Whitney U test were used to compare parametric and nonparametric continuous variables, respectively. Healthy volunteers were enrolled to create a matched control group. Categorical variables were compared by Chi-square (χ^2^) test. A two-tailed p-value of <0.05 was considered statistically significant. All data were analyzed using SPSS v23.0 (IBM Corp, Armonk, NY, USA).

## Results

From February 2020 to December 2021, 150 patients were diagnosed with left ventricular hypertrophy. After exclusion, 36 patients with HCM were included in the study. The control group comprised 34 individuals who were referred for several reasons for CMR and were found to have normal CMR scans at the end of the assessment. The mean age of the HCM population was 43.2 + 13.5 years, while it was 37.5 + 11.3 in the control group. The female ratio of the HCM group was found to be 36%, while it was 56% in the healthy control group. The basic characteristic features of the patients are given in Table 1.

**Table 1:** Characteristic features of HCM and control group CMR: cardiac magnetic resonance, BMI: body mass index, BSA: body surface area, HCM: hypertrophic cardiomyopathy. "Gender" parameter was analyzed by the Chi-Square test, while the other parameters (mean ± standard deviation) were compared using Student t-test between groups.

CMR Parameters	Control (n=34)	HCM (n=36)	p-value
Age (y)	37.5 ± 11.3	43.2 ± 13.5	0.060
Gender	19 (56%)	13 (36%)	0.149
BSA (m^2^)	1.93 ± 0.2	1.98 ± 0.3	0.418
BMI (ml/m2)	22.3 ± 2.2	23.3 + 2.6	0.088

MAPSE values were significantly higher in the normal control group when compared to the HCM patients (MAPSE: 14.5 ± 2.9 mm vs. 11.7 ± 3.2 mm; p<0.001), while TAPSE values did not show a significant difference between the two groups (p=0.627) (Table 2). Another significant difference between the HCM and control group was basal anteroseptal wall thickness as expected considering the definition of HCM. The mean basal anteroseptal segment was 15.5 ± 5.7 mm in the HCM group, while it was 8.4 ± 2 mm in the control group (p<0.001). LV end-systolic diameter was also significantly smaller in the HCM group (21.7 ± 2.8 mm vs. 32.7 ± 3.5 mm, p<0.001). Another significant result of the two patient groups was the left atrium (LA) area. The mean LA area in the HCM group was significantly greater than that of the normal control group (26.4 ± 9.2 cm^2^ vs. 20.6 ± 3.2 cm^2^, p<0.001). All the rest of the measurements are given in Table 2.

**Table 2:** CMR parameters of HCM and control group CMR: cardiac magnetic resonance, HCM: hypertrophic cardiomyopathy, EDD: end-diastolic diameter, EDVI: end-diastolic volume index, EF: ejection fraction, ESD; end-systolic diameter, ESVI: end-systolic volume index, LA: left atrium, LV: left ventricle, MAPSE: mitral annular plane systolic excursion, RA: right atrium, RV: right ventricle, TAPSE: tricuspid annular plane systolic excursion. All continuous parameters (mean ± standard deviation) were compared using Student t-test between groups.

CMR Parameters	Control (n=34)	HCM (n=36)	p-value
LVEDD (mm)	47.9 ± 4.2	48.4 ± 6.3	0.682
LVESD (mm)	32.7 ± 3.5	21.7 ± 2.8	<0.001
Anteroseptum basal segment (mm)	8.4 ± 2	15.5 ± 5.7	<0.001
Inferolateral basal segment (mm)	6.7 ± 1.6	7.6 ± 2.3	0.095
LVEDVI (ml/m^2^)	74.5 ± 11.1	74.1 ± 12.9	0.885
LVESVI (ml/m^2^)	28.2 ± 5.7	27.5 ± 10.4	0.718
RVEDVI (ml/m^2^)	68.7 ± 12.4	74.6 ± 14.3	0.071
RVESI (ml/m^2^)	27 ± 7.5	30.4 ± 8.2	0.076
LVEF (%)	63.5 ± 8.2	62.5 ± 3.2	0.510
RVEF (%)	62.1 ± 6.3	59.6 ± 5.4	0.078
LA area (cm^2^)	20.6 ± 3.2	26.4 ± 9.2	0.001
RA area (cm^2^)	21.2 ± 5.7	18.7 ± 4.9	0.054
TAPSE (mm)	21.1 ± 4.2	20.6 ± 4.2	0.627
MAPSE (mm)	11.7 ± 3.2	14.5 ± 2.9	<0.001

## Discussion

The main finding in our study was that the MAPSE value showing left ventricular longitudinal functions was lower in the HCM group compared to the control group. Unlike MAPSE, there was no significant difference between the two groups in TAPSE values. MAPSE is a parameter that can be easily evaluated from the septal or lateral part of the left ventricle by echocardiography in routine clinical practice and is correlated with the systolic longitudinal functions of the left ventricle. Although there is no clear opinion regarding the region from where the measurement gives better results, the lateral region is generally preferred in daily practice [11,13]. We also routinely perform lateral MAPSE measurement in our own clinic, and we used these data in our study.

Apart from echocardiography, MAPSE also gives very good results when evaluated with CMR, and the intra- or inter-observer differences are very low compared to echocardiography [14]. This value, which shows the movement of the mitral valve annulus in the long axis, has been shown to play a fundamental role in the mechanics of the heart in recent years and is thought to be an early indicator of many pathological conditions [15,16]. The contribution of long-axis function to the overall stroke volume has been evaluated using CMR in healthy individuals, athletes and patients with dilated cardiomyopathies. These studies demonstrate that long-axis functions contribute around 60% to stroke volume [16].

A multicenter study in patients with hypertension who underwent CMR for different indications revealed that lateral MAPSE measurement evaluated by CMR was an independent predictor of mortality. Each 1 mm reduction in the lateral MAPSE was associated with a 40.2% increased risk of death after adjusting for clinical and imaging risk factors. Another important finding in this study is that lateral MAPSE is a strong independent prognostic marker even in hypertensive patients with preserved ejection fraction or without a history of myocardial infarction, and it has demonstrated that it potentially allows early identification of patients at the highest risk [17].

In echocardiography studies of patients with hypertension, it has been shown that the MAPSE value decreases significantly despite preserved left ventricular functions [18,19]. In HCM, like in patients with hypertension, the responsible pathophysiological mechanisms mainly lead to diastolic dysfunction, and diastolic dysfunction is traditionally preserved and sometimes even with an ejection fraction above normal in HCM [20]. Diastolic dysfunction is one of the early symptoms of hypertrophic cardiomyopathy and hypertensive heart disease, which can be subclinical, and is an important cause of mortality and morbidity in the later stages [21]. This may also provoke deterioration in systolic longitudinal functions, which is associated with a poor prognosis in the later stages of the disease [22].

Although high atrial and ventricular diastolic pressures are generally evaluated by echocardiographic Doppler measurements, E/e' and left atrial volume index (LAVI) parameters, which are also used in the diagnosis of preserved heart failure, were found to be correlated with MAPSE systolic functions [23]. In several speckle tracking echocardiography (STE) studies, there is a decrease in left ventricular longitudinal function in the early stages of HCM [24]. Considering that CMR is frequently performed both for the initial diagnosis and prognosis, MAPSE measurement can be utilized as an easy and reliable method in this patient group as an early indicator of both diastolic and systolic longitudinal dysfunction, which deteriorates later in the disease. It may also be a marker of left ventricular fibrosis, which is a very robust prognostic factor. In the study of Doesch et al., it was found that when LGE, which is one of the important indicators of prognosis in patients with HCM increased, the MAPSE value decreased which was associated with adverse clinical events [14].

We found in our study that the lateral MAPSE values in patients with HCM were lower than the ones in the healthy control group. Although the septum is generally affected in HCM, we think that the lower lateral MAPSE values compared to the normal group are important indicators of diastolic dysfunction in this patient group. Similar to MAPSE, it is known that right ventricular functions are adversely affected in HCM patients, and the TAPSE value is low in both echocardiographic Doppler studies and CMR studies, which are the gold standard in RV evaluation [25,26]. In our study, although TAPSE values were lower in the HCM group, there was no statistical significance. We think that the reason for this is related to the low number of patients.

One of the important results of our study was that the left atrium area was higher in the HCM group. Many factors such as diastolic dysfunction, atrial myopathy and mitral regurgitation are responsible for left atrial enlargement in patients with HCM [27], and patients with left atrial enlargement have significant adverse effects such as atrial fibrillation, stroke, sudden death and congestive heart failure compared to patients without left atrial enlargement. The risk of experiencing cardiac and cardiovascular events is higher [28].

Study limitations

The limitations of this study follow from its retrospective single-center, cross-sectional analysis study design, which might have caused selection bias. As patients were referred for CMR studies to our center, a comparison of detailed echocardiography was not possible for each patient. Additionally, given the progressive course of HCM, a follow-up CMR study would have been designed as well. Larger studies with a precise follow-up to assess clinical outcomes and comparison of multi-modality assessments are needed.

## Conclusions

In conclusion, we demonstrated that MAPSE values as robust measures of left ventricle longitudinal function are significantly lower in the HCM patient group when compared to the healthy control group. Similarly, TAPSE values as important measures of right ventricle longitudinal function were lower in the HCM patient group when compared to the control group, yet with no statistical significance. There is a need for further studies for the verification of these results in larger cohorts.
